# Radiomics and machine learning applied to STIR sequence for prediction of quantitative parameters in facioscapulohumeral disease

**DOI:** 10.3389/fneur.2023.1105276

**Published:** 2023-02-24

**Authors:** Giulia Colelli, Leonardo Barzaghi, Matteo Paoletti, Mauro Monforte, Niels Bergsland, Giulia Manco, Xeni Deligianni, Francesco Santini, Enzo Ricci, Giorgio Tasca, Antonietta Mira, Silvia Figini, Anna Pichiecchio

**Affiliations:** ^1^Department of Mathematics, University of Pavia, Pavia, Italy; ^2^Neuroradiology Department, Advanced Imaging and Radiomics Center, IRCCS Mondino Foundation, Pavia, Italy; ^3^INFN, Group of Pavia, Pavia, Italy; ^4^UOC di Neurologia, Fondazione Policlinico Universitario A. Gemelli IRCCS, Rome, Italy; ^5^Department of Neurology, Jacobs School of Medicine and Biomedical Sciences, Buffalo Neuroimaging Analysis Center, University of Buffalo, The State University of New York, Buffalo, NY, United States; ^6^IRCCS, Fondazione Don Carlo Gnocchi ONLUS, Milan, Italy; ^7^Department of Radiology, University Hospital Basel, Basel, Switzerland; ^8^Basel Muscle MRI, Department of Biomedical Engineering, University of Basel, Basel, Switzerland; ^9^John Walton Muscular Dystrophy Research Centre, Newcastle University and Newcastle Hospitals NHS Foundation Trusts, Newcastle upon Tyne, United Kingdom; ^10^Data Science Lab, Università della Svizzera italiana, Lugano, Switzerland; ^11^Department of Science and High Technology, University of Insubria, Como, Italy; ^12^Department of Political and Social Sciences, University of Pavia, Pavia, Italy; ^13^BioData Science Center, IRCCS Mondino Foundation, Pavia, Italy; ^14^Department of Brain and Behavioural Sciences, University of Pavia, Pavia, Italy

**Keywords:** radiomics, machine learning, muscle MRI, stir, FSHD

## Abstract

**Purpose:**

Quantitative Muscle MRI (qMRI) is a valuable and non-invasive tool to assess disease involvement and progression in neuromuscular disorders being able to detect even subtle changes in muscle pathology. The aim of this study is to evaluate the feasibility of using a conventional short-tau inversion recovery (STIR) sequence to predict fat fraction (FF) and water T2 (wT2) in skeletal muscle introducing a radiomic workflow with standardized feature extraction combined with machine learning algorithms.

**Methods:**

Twenty-five patients with facioscapulohumeral muscular dystrophy (FSHD) were scanned at calf level using conventional STIR sequence and qMRI techniques. We applied and compared three different radiomics workflows (*WF1, WF2, WF3*), combined with seven Machine Learning regression algorithms (linear, ridge and lasso regression, tree, random forest, k-nearest neighbor and support vector machine), on conventional STIR images to predict FF and wT2 for six calf muscles.

**Results:**

The combination of WF3 and K-nearest neighbor resulted to be the best predictor model of qMRI parameters with a mean absolute error about ± 5 *pp* for FF and ± 1.8 *ms* for wT2.

**Conclusion:**

This pilot study demonstrated the possibility to predict qMRI parameters in a cohort of FSHD subjects starting from conventional STIR sequence.

## 1. Introduction

Muscle Magnetic Resonance Imaging (mMRI) has been increasingly used over the last years as a powerful diagnostic tool to evaluate disease involvement and progression in several neuromuscular disorders (1–3). mMRI is able to demonstrate selective patterns of damage distribution both in terms of fat replacement and muscular edema (4, 5). Facioscapulohumeral muscular dystrophy (FSHD) is a genetic muscle disorders that causes a slowly progressive and asymmetric weakness of the facioscapulohumeral, abdominal, paraspinal, and lower leg muscles (6–9) both in pediatric and adult patients. mMRI of FSHD has relied on acquisition of conventional sequences such as T1-weighted (T1w) and short-tau inversion recovery (STIR) sequences that are able to foster the qualitative detection of anatomical changes in muscles size or shape, particularly related to fat replacement and muscle edema (or edema –like) (10, 11), revealing a widespread involvement both in upper girdle and lower limbs (12, 13). The use of mMRI enabled to propose a peculiar model for FSHD disease evolution, highlighting how patients undergo a muscle-selective involvement with an early hyperintense signal on STIR sequence related to edema/inflammation, followed by fatty replacement of single muscles, particularly evident on T1w images (14). Recently, the use of STIR signal intensity as a longitudinal marker of inflammation suppression in FSHD has been questioned because an incremental STIR signal has been reported in FSHD patients during the immunosuppressive treatment period (15). As per other neuromuscular diseases, semi-quantitative visual scales have been applied to support and improve the evaluation of morphological changes in muscles, e.g., Mercuri and Fischer scales (16, 17). The recent development and implementation of quantitative MRI (qMRI) in the field of neuromuscular diseases allowed to go beyond the conventional and semi-quantitative approaches, being able to assess quantitative parameters (e.g., the percentage of fat replacement in the muscle, the so called fat fraction, FF), that have been correlated both with transcriptome signatures (DUX4 and PAX7 signatures) and with clinical tests (e.g., Ricci clinical severity score) (18). Therefore the development of qMRI techniques improved the non-invasive applicability of muscle imaging in the diagnostic process and follow-up of muscle disorders (19). Neither the clinical outcomes nor the conventional muscle MRI techniques, in fact, are deemed to be sensitive enough to track muscle changes in slowly progressing diseases (3). qMRI is considered a valuable tool to monitor even fine changes in neuromuscular disease evaluation and longitudinal progression over time because it delivers quantitative information such as muscles FF and the muscle water T2 (wT2) relaxation time which is an unspecific marker for disease activity because it is sensitive to the presence of leaky membranes, muscle fiber necrosis, edema, inflammation, or denervation (20). Dixon imaging and Multi-Echo T2 spin-echo sequences are the most commonly used qMRI methods to compute FF and wT2 (3). Up-to-date qMRI methods require custom-tailored sequences provided by vendors on the MRI scanner resulting in high-cost implementations. Recently, Image Biomarker Standardization Initiative (IBSI, https://ibsi.readthedocs.io/en/latest/) radiomics proved to be a powerful tool to extract quantitative information from MRI images, becoming a new asset in the diagnostic field (21). It can identify the main patterns of a disease through the mathematical extraction of pixels intensity and spatial interrelationships distributions. Radiomics quantifies textural information that, once dimensionally reduced (22, 23), can be combined with machine learning (ML) algorithms to predict neuromuscular quantitative biomarkers such as FF and wT2 with a good predictive power (24). Standardized features extraction can also help to overcome possible limitations due to the presence of fat in the evaluation of wT2 biomarkers through exponential fitting. However, it is still unclear whether and how radiomics could be applied on conventional STIR images and combined with ML algorithms to predict FF and wT2. Moreover, it remains unexplored whether the predictive power of ML algorithms on conventional STIR images could be improved through the definition of new radiomic features as an alternative to the ones provided by commercial radiomic features extraction software (25).

STIR sequence is most likely available in all MRI centers and it has a very competitive acquisition time compared to qMRI sequences. In this study, we aim to investigate whether different radiomics and machine learning algorithms may be applied to conventional STIR sequence to predict quantitative parameters in skeletal muscle.

## 2. Materials and methods

**Figure 1:**
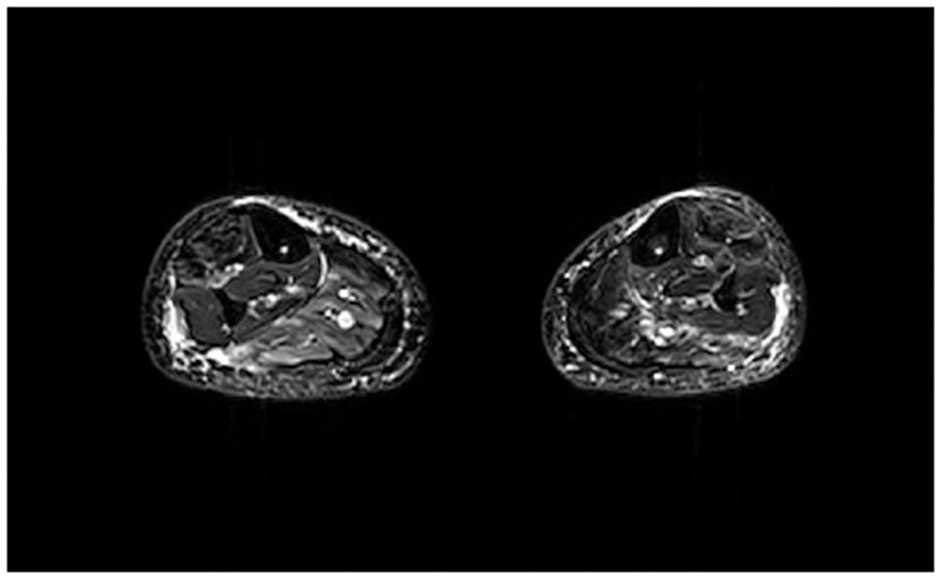
Example of axial STIR image of an FSHD subject at calf level. Image acquired at Neuroradiology Department of IRCCS Mondino Foundation.

Twenty-five FSHD patients (10 females, age range: 19–60 y) and six healthy volunteers (HCs) (5 females, age range: 47–63 y) were scanned on a 3T MRI scanner (Magnetom Skyra, Siemens Healthcare, Erlangen, Germany) using integrated spine and body surface coils. Acquisition volume was centered on the calf with the last acquired slice located at 6 cm proximally from the upper limit of the patella. The MRI protocol included 3D 6-point multi-echo gradient-echo (MEGE) [52 slices, slice thickness = 5.0 mm, distance factor = 20%, resolution = 1 × 1 × 5 mm3, TR/ TE = 35 ms/1.7–9.2 ms, scan time = 15 min], multi-echo spin echo (MESE) [7 slices, TH = 10 mm, DF = 300%, resolution = 1.2 × 1.2 × 10 mm3, TR/TE = 4,100 ms/10.9–185.3 ms, 17 echoes, scan time = 5.13 min] and 2D STIR sequences [50 slices, TH = 5.0 mm, DF = 20%, resolution = 1 × 1 × 5mm3, TR/TE = 4,200/82 ms, TI = 230 ms, scan time = 3.40 min]. An example of STIR image is reported in Figure 1. Pre-processing steps have been performed on STIR images in order to ensure features extraction on an inter-patients harmonized grayscale values. In particular, all images were pre-processed by 3DSlicer (26) N4 Bias Field Correction to correct low frequency intensity non-uniformity in MRI images, and 3DSlicer Histogram Matching to normalize grayscale MRI images.

**Figure 2:**
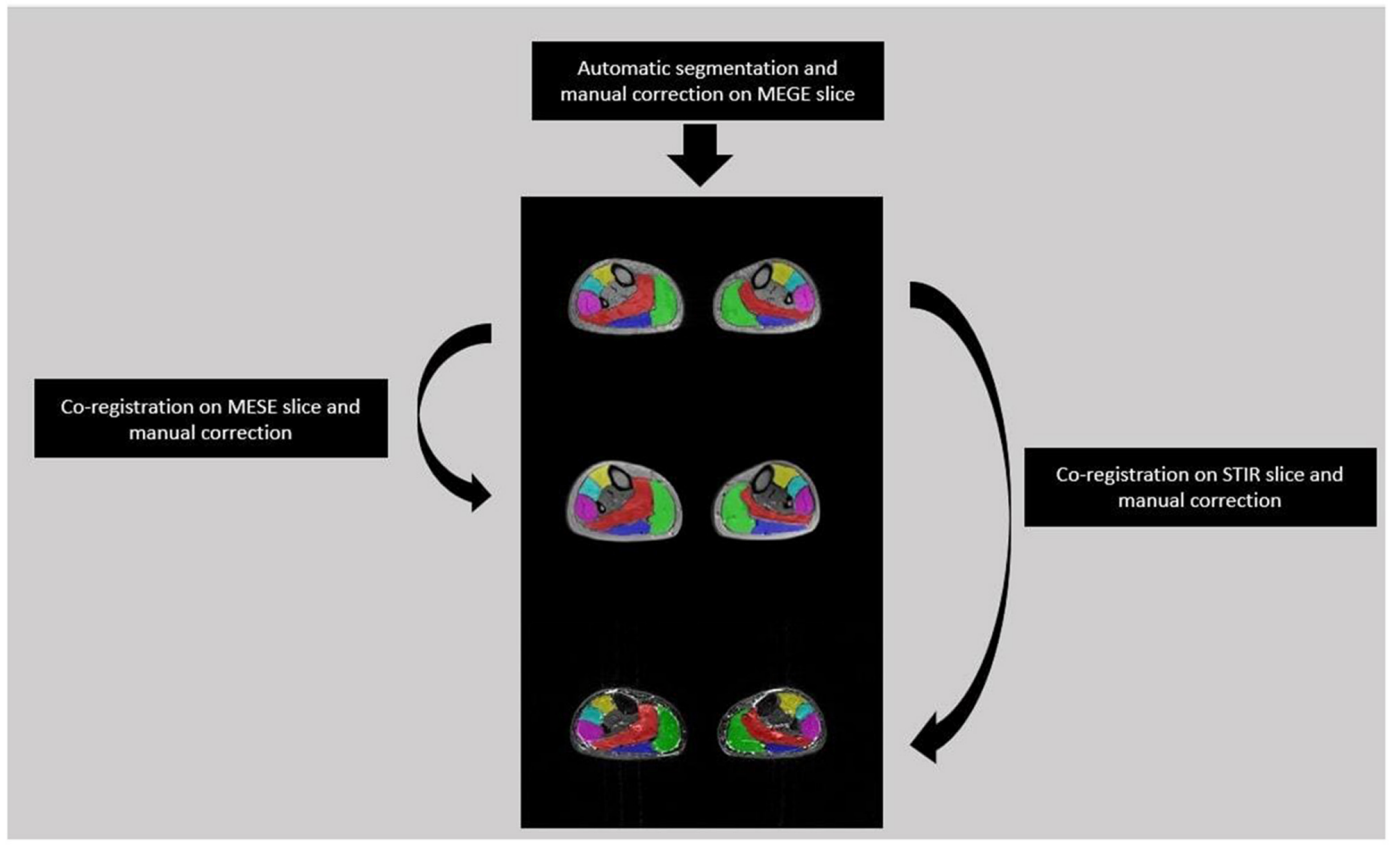
Segmentation flow from MEGE to MESE and STIR images. Automatic segmentation was performed on MEGE slice followed by manual correction for 6 ROIs: Soleus (Red), Medial and Lateral Gastrocnemius (Green, Dark Blue), Anterior Tibialis (Yellow), Extensor Digitorum Longus (Light Blue), Peroneus Longus (Pink). Then the ROIs were co-registered and manually corrected both on MESE and STIR images.

A single slice from the medial calf level of each FSHD patient was selected from the first echo images of MEGE because of the higher SNR than the other echoes. Each selected slice was automatically segmented (27) into six regions of interest (ROIs) for each calf muscle, i.e. Soleus (S), Medial and Lateral Gastrocnemius (MG, LG), Anterior Tibialis (TA), Extensor Digitorum Longus (ELD), Peroneus Longus (Pe). The ROIs were co-registered to the medial calf slice of MESE and STIR using the linear registration command ‘flirt' of FSL software (28). A single trained operator with 3 years of experience manually corrected each ROIs after the automatic segmentation of MEGE images and after the co-registration on MESE and STIR images (Figure 2).

For each subject and each muscle, radiomic features extraction and ML prediction were performed on the mid-calf slice of STIR image because it gives a representation of all calf muscles with a cross sectional area (CSA) wide enough to ensure the extraction of a robust pixel intensity distribution (29). Fifty six radiomics features were extracted averaging left and right side per each muscle. In particular, we extracted 25 first-order statistical-based features concerning voxels intensity distributions, e.g., CONVENTIONAL_mean, CONVENTIONAL_std, CONVENTIONAL_max, CONVENTIONAL_Q1, 26 second-order statistical-based features highlighting voxels spatial relationship such as the gray level co-occurrence matrix (GLCM) features (e.g., GLCM_Correlation, GLCM_Entropy_log10) and the gray level zone length matrix (GLZLM) features (e.g. GLZLM_LZE, GLZLM_LGZE, GLZLM_HGZE), 5 shape related features concerning size and geometric properties (e.g. SHAPE_Volume(mL), SHAPE_Volume(vx)) (25). Finally, ground truth FF and wT2 values, which the ML predictions have been compared to, were calculated by Fatty Riot algorithm (30) and by EPG signal simulation (two-component model, both for water and fat) (31, 32) from mid-calf MEGE and MESE slice, respectively.

### 2.1. Dataset, dimensionality reduction, and machine learning algorithms

We compare the performance in predicting calf muscle FF and wT2 values introducing three different workflows. In particular, inspired by Felisaz et al. (24) work, the first workflow predicts FF and wT2 combining radiomics with LIFEx software (25), principal component analysis (PCA) (33) and ML regression models. The second method uses the same features extraction and ML models of the previous method but explores the use of a new dimensionality reduction technique (23) as an alternative to PCA to verify a possible improvement in the prediction of neuromuscular quantitative parameters. The third method relies neither on LIFEx features nor on any dimensionality reduction technique. In particular, two STIR-based features are defined as markers of muscle fat percentage and muscle inflammation. These two features are used as predictors in ML models to test whether there is an improvement in the predictive performance of FF and wT2.

### 2.2. Workflow 1

Features extraction was performed using the IBSI standard-compliant LIFEx software v.7.1.0 with the aim to extract shape related features, taking into account for size and geometric properties, first-order statistical-based features, concerning voxels intensity distributions and second-order statistical-based features highlighting voxels spatial relationship. In particular, a 2D extraction was performed on each ROI corresponding to the six calf muscles (left and right side were averaged). Therefore, we obtained six datasets associated with each calf muscle. On each dataset principal component analysis (PCA) (33) dimensional reduction was performed in order to obtain lower-dimensional data while preserving as much of the data variation as possible. Six principal components, which in our case retain about 90% of the explained variance, were identified and consequently each data point was projected onto them. For each muscle dataset we implemented the parametric linear (34), ridge (35) and Lasso (36) regression and the non-parametric KNN (37), SVM (38), tree (39), and RF (40) algorithms. A k-fold cross validation resampling approach with *k* = 5 was used on the associated PCA dimensionally reduced dataset. This procedure guarantees a more realistic performance evaluation of each machine learning model by fitting the same statistical model several times on randomly obtained subsets of approximately equal size.

### 2.3. Workflow 2

**Figure 3:**
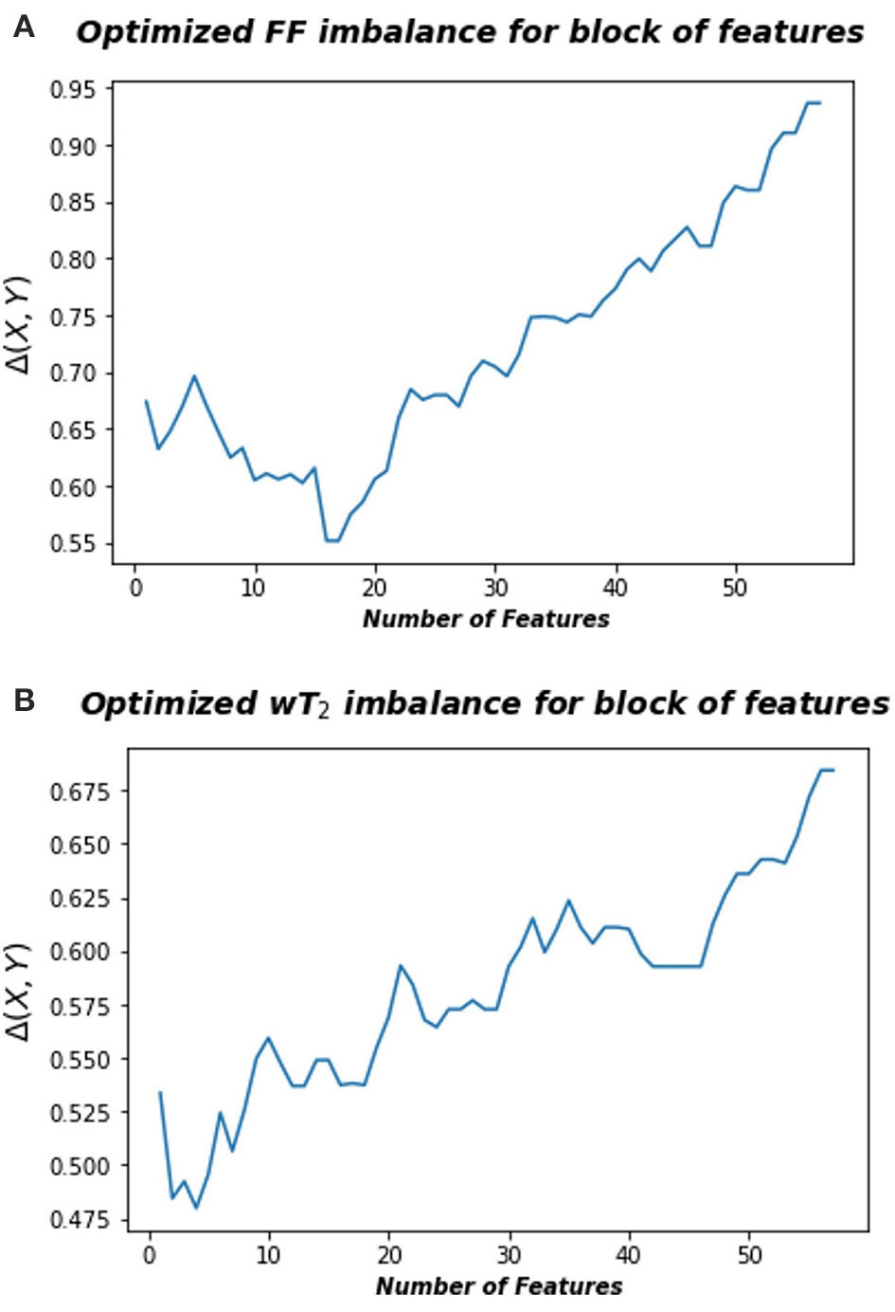
Optimized information imbalance for blocks of features for the Soleus muscle. On the y-axis are reported the optimized information imbalance values, which are calculated using Equation (1), as a function of subsets of radiomic features (x-axis). **(A)** Optimized imbalance with respect to the target biomarker FF (top) and **(B)** to the wT2 (bottom).

The starting point was the 2D extraction of texture features from the pre-processed STIR image as described in *WF1*. To reduce the dimensionality of the dataset we have used the concept of information imbalance described in Glielmo et al. (23). More precisely, performing feature selection or dimensionality reduction in our case is the same task of finding the most suitable measure between data points since explicit features are available. This is because a particular choice of features naturally gives rise to a different distance function computed through the Euclidean norm (23). Therefore, we designed a feature selection algorithm by selecting the subset of features, which minimizes the information imbalance with respect to the two targets, the values of the neuromuscular biomarkers FF and wT2, separately. The definition of information imbalance Δ used was its estimation on a dataset with N points (23):


(1) $$\begin{array}{l} \Delta \left(A\to B\right)\approx \frac{2\left(r^{B}|\;r^{A}=1\right)}{N} \end{array}$$

where A is the space consisting in the radiomic feature space and B is the space associated to FF or wT2 biomarkers, *r*^*B*^ and *r*^*A*^ represent the rank of each pair points in the space B and A, respectively, calculated according to the distance *d*_*B*_ and *d*_*A*_, an euclidean norm defined in the relative space. Thus, information imbalance quantifies the relative information content of a distance measure with respect to another using the widespread idea of local neighborhoods. A low value of Δ (*A*→*B***)** means that the combination of certain features can predict a specific neuromuscular biomarker. Figure 3 shows for Soleum the minimum information imbalance Δ (*A*→*B*) achievable with a specific subset of radiomics features for the two biomarkers wT2 and FF. For each muscle, we optimized the information imbalance with respect to target FF and wT2 separately and selected the subspace of radiomics features corresponding to the associated minimum Δ. The obtained datasets for each muscle and each biomarker were used as input for machine learning algorithms. As in *WF1* parametric and non-parametric algorithms were implemented using the resampling k-folds cross validation.

### 2.4. Workflow 3

We defined two STIR-based radiomic features to be used as an alternative to the conventional textural features of WF1 and WF2. We use these new features as the only covariates in the implementation of ML algorithms to test whether the prediction performance of ML models could be improved over those obtained by the previously described workflows. Firstly, we applied the same segmentation method of FSHD patients on the pre-processed STIR images of each healthy control (HC). In particular, six contiguous HCs slices of mid-calf region were segmented in order to ensure a robust pixel statistics of the grayscale intensity distributions. Then, two reference limits, Upper Limit (UL) and Lower Limit (LL), were defined as follows. Inspired by Dahlqvist et al. (41), UL was defined for each calf muscle through the extraction of a pixel-wise histogram of signal intensity distribution from all slices. The six muscle-wise UL were set at the mean μ of the associated pixels-intensity distribution added to 2 standard deviation (S.D.) σ:


(2) $$\begin{array}{l} {UL}_{i}=\mu_{i}+2\sigma_{i} \end{array}$$

with *i* indexing the six calf muscles.

Due to non-uniform fat suppression of STIR sequence, LL was calculated as a representative value of fat signal intensity. Therefore, subcutaneous fat (average thickness at medial level of HCs was about 10.5 mm) was manually drawn in HCs slices to ensure the extraction of LL feature. In particular, from subcutaneous fat ROI of all slices the pixel-wise histogram of signal intensity distribution was extracted. Subsequently, the LL was set as the mode of the distribution. In this way, we could calculate a more realistic fat intensity representative value, limiting the contribution of blood vessels present in the subcutaneous fat, which tend to shift the mean value of the associated distribution toward greater value due to the hyperintesity STIR signal of the blood.

Moreover, the obtained LL and muscle-wise UL coefficients were set as the reference limits to quantify, for every FSHD patient, fat infiltration grade (FFG) and muscle edema grade (MEG) by expressing the number of pixels below LL and above UL as a percentage of the total pixels in each calf muscle. FFG and MEG were then used as covariates in ML models to predict FF and wT2, respectively. Particularly, muscle-wise FFG and MEG values were separately collected into datasets according to calf muscles and neuromuscular biomarker and used as input for machine learning algorithms.

As described in *WF1*, we implemented both parametric and non-parametric models using the k-folds cross validation as a resampling approach. *WF3* brought the advantage of testing the prediction accuracy of neuromuscular biomarkers with two features that were easy to compute by means of a stand-alone Python routine, without going through commercial texture software and any dimensionality reduction techniques.

### 2.5. ML models performance evaluation

According to the aforementioned workflows, models performance estimation was performed calculating for each muscle and for each ML algorithm the mean absolute error (MAE):


(3) $$\begin{array}{l} {MAE}_{j}=\frac{\Sigma_{i=1}^{N}|y_{i}-{\bar{y}}_{i}\;|}{N} \end{array}$$

where *N* is the number of observations, *y*_*i*_ is the target value, ȳ_*i*_ the predicted value, index *j* is related to the different calf muscles and index *i* runs over the observations associated with each muscle. Furthermore, mean MAE ($\bar{MAE}$) was defined as:


(4) $$\begin{array}{l} {\bar{MAE}}_{j}=\frac{\Sigma_{k=1}^{5}{MAE}_{j}}{k} \end{array}$$

where the index *k* runs over the *k* = 5 folds.

To measure the variability of volume and ground truths distribution we also calculated the coefficients of variation (CVs) defined as:


(5) $$\begin{array}{l} CV_{i}=\frac{\sigma_{i}}{\mu_{i}} \end{array}$$

where the index *i* runs over the muscles, σ_*i*_ and μ_*i*_ are the associated S.D. and mean of the distributions, respectively. Thus, CVs for volume and ground truth muscle-wise FF and wT2 quantify the variability range of ground truth values on which the ML models were tested.

Moreover, we explored whether $\bar{MAE}\;$prediction shows linear or monotonic dependency on CV values of muscle volume and ground truth parameters using Pearson (ρ_*P*_) and Spearman (ρ_*S*_) correlation coefficients.

## 3. Results

**Figure 4:**
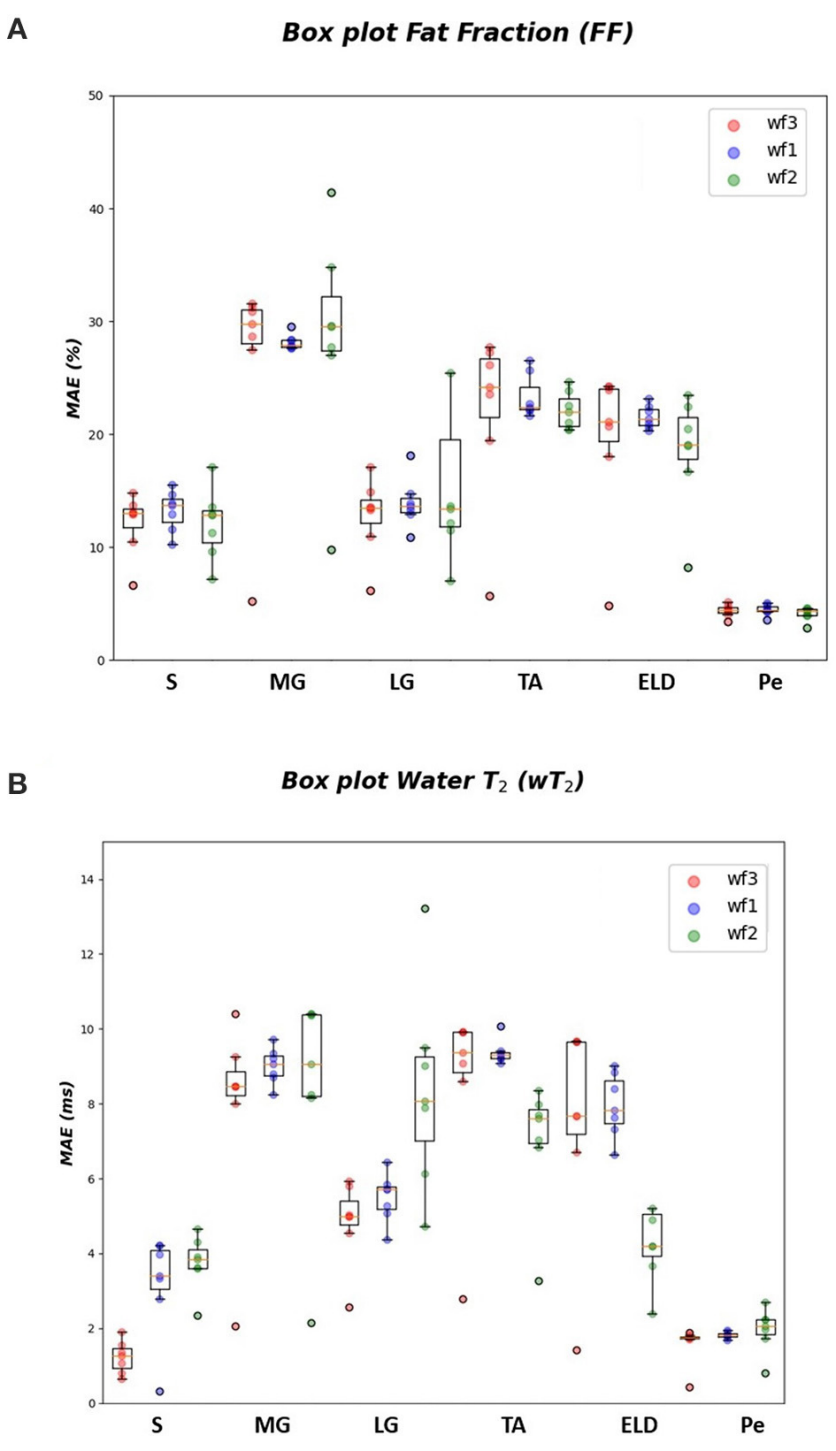
FF and wT2 boxplots. Muscle-wise boxplots (first quartile (Q1) to third quartile (Q3) and median value in orange line) **(A)** for FF (top) expressed in percentage points (pp) and **(B)** wT2 (bottom) expressed in ms. Three boxplots are given for each muscle related to WF 1 (blue), WF 2 (green), WF 3 (red). Highest accuracy is related to red dots (FF, wT2 boxplots) corresponding to KNN prediction performances.

In Tables 1–3 the FF $\bar{MAE}$ was reported for the three used workflows (*WF1, WF2, and WF3*) calculated for each muscle and from each ML algorithm. Similarly, in Tables 4–6 the $\bar{MAE}$ was reported for wT2. Boxplots in Figure 4 show the FF and wT2 $\bar{MAE}$ distribution per each muscle and workflow (*WF 1, 2*, and *3*). The discrepancy between the ground truth values and ML predicted values are expressed in percentage points (pp) for FF and in milliseconds (ms) for wT2, respectively.

**Table 1:** Workflow 1: Evaluation of ML models predicting performances: mean absolute discrepancy ($\bar{MAE}$) between the muscle-wise Fat Fraction gold standard values from Fatty Riot algorithm and the predicted value through ML algorithms.

**Mean absolute discrepancy (** $\bar{MAE}$ **)**							
**Muscle**	**LR**	**Ridge**	**Lasso**	**TREE**	**RF**	**KNN**	**SVM**
S	0.155 (0.052)	0.139 (0.047)	0.130 (0.042)	0.147 (0.037)	0.137 (0.035)	0.116 (0.064)	0.102 (0.058)
MG	0.284 (0.064)	0.283 (0.059)	0.295 (0.054)	0.276 (0.068)	0.278 (0.073)	0.279 (0.066)	0.276 (0.066)
LG	0.066 (0.074)	0.133 (0.032)	0.139 (0.036)	0.129 (0.027)	0.147 (0.030)	0.137 (0.032)	0.109 (0.034)
TA	0.225 (0.039)	0.220 (0.039)	0.247 (0.051)	0.239 (0.035)	0.205 (0.013)	0.204 (0.030)	0.210 (0.030)
ELD	0.225 (0.028)	0.191 (0.021)	0.235 (0.0334)	0.205 (0.018)	0.189 (0.028)	0.082 (0.010)	0.167 (0.028)
Pe	0.039 (0.02)	0.046 (0.01)	0.043 (0.011)	0.044 (0.017)	0.046 (0.0117)	0.028 (0.011)	0.039 (0.017)

S.D. is reported in round brackets.

**Table 2:** Workflow 2: Evaluation of ML models predicting performances: mean absolute discrepancy ($\bar{MAE}$) between the muscle-wise Fat Fraction gold standard values from Fatty Riot algorithm and the predicted value through ML algorithms.

**Mean absolute discrepancy (** $\bar{MAE}$ **)**							
**Muscle**	**LR**	**Ridge**	**Lasso**	**TREE**	**RF**	**KNN**	**SVM**
S	0.171 (0.090)	0.135 (0.050)	0.130 (0.042)	0.128 (0.053)	0.113 (0.063)	0.072 (0.035)	0.096 (0.054)
MG	0.414 (0.180)	0.271 (0.052)	0.296 (0.053)	0.348 (0.042)	0.295 (0.051)	0.098 (0.033)	0.277 (0.050)
LG	1.133 (1.967)	0.255 (0.253)	0.136 (0.038)	0.121 (0.031)	0.134 (0.058)	0.134 (0.032)	0.115 (0.043)
TA	0.225 (0.039)	0.220 (0.039)	0.247 (0.051)	0.239 (0.035)	0.204 (0.013)	0.204 (0.030)	0.210 (0.030)
ELD	0.225 (0.028)	0.191 (0.021)	0.237 (0.033)	0.205 (0.0178)	0.189 (0.028)	0.082 (0.010)	0.167 (0.028)
Pe	0.039 (0.020)	0.046 (0.009)	0.043 (0.011)	0.044 (0.017)	0.046 (0.012)	0.028 (0.011)	0.039 (0.017)

S.D. is reported in round brackets.

**Table 3:** Workflow 3: Evaluation of ML models predicting performances: mean absolute discrepancy ($\bar{MAE}$) between the muscle-wise Fat Fraction gold standard values from Fatty Riot algorithm and the predicted value through ML algorithms.

**Mean absolute discrepancy (** $\bar{MAE}$ **)**							
**Muscle**	**LR**	**Ridge**	**Lasso**	**TREE**	**RF**	**KNN**	**SVM**
S	0.130 (0.028)	0.130 (0.031)	0.130 (0.036)	0.137(0.032)	0.148 (0.043)	0.066 (0.031)	0.105 (0.054)
MG	0.312 (0.041)	0.309 (0.034)	0.297 (0.021)	0.286 (0.064)	0.275 (0.047)	0.052 (0.012)	0.316 (0.054)
LG	0.135 (0.030)	0.135 (0.030)	0.134 (0.030)	0.149 (0.018)	0.171 (0.026)	0.061 (0.012)	0.110 (0.023)
TA	0.277 (0.043)	0.273 (0.037)	0.262 (0.035)	0.242 (0.068)	0.235 (0.078)	0.057 (0.012)	0.194 (0.062)
ELD	0.242 (0.040)	0.242 (0.039)	0.240 (0.035)	0.270 (0.051)	0.211 (0.059)	0.048 (0.019)	0.180 (0.051)
Pe	0.045 (0.019)	0.044 (0.019)	0.044 (0.020)	0.048 (0.021)	0.052 (0.020)	0.034 (0.019)	0.040 (0.024)

S.D. is reported in round brackets.

**Table 4:** Workflow 1: Evaluation of ML models predicting performances: mean absolute discrepancy ($\bar{MAE}$ expressed in ms) between the muscle-wise water T2 gold standard values from EPG signal simulation algorithm and the predicted value through ML algorithms.

**Mean absolute discrepancy (** $\bar{MAE}$ **)**							
**Muscle**	**LR**	**Ridge**	**Lasso**	**TREE**	**RF**	**KNN**	**SVM**
S	4.21 (0.52)	4.21 (0.55)	3.98 (0.65)	3.33 (1.23)	2.78 (0.68)	3.40 (0.87)	0.32 (0.81)
MG	9.22 (1.90)	9.05 (1.81)	8.80 (1.77)	9.73 (1.68)	9.35 (2.61)	8.72 (2.11)	8.25 (2.61)
LG	6.44 (2.49)	5.71 (1.29)	5.07 (0.39)	5.84 (1.46)	5.71 (1.59)	5.28 (0.730)	4.38 (1.68)
TA	9.30 (2.42)	9.22 (2.38)	9.09 (2.50)	9.34 (3.11)	10.08 (3.48)	9.42 (3.44)	9.22 (2.83)
ELD	9.03 (4.13)	8.83 (3.97)	8.41 (3.59)	7.33 (2.08)	7.83 (3.09)	7.64 (3.05)	6.64 (2.93)
Pe	1.96 (0.472)	1.92 (0.413)	1.83 (0.325)	1.83 (0.384)	1.68 (0.25)	1.81 (0.33)	1.76 (0.20)

S.D. is reported in round brackets.

**Table 5:** Workflow 2: Evaluation of ML models predicting performances: mean absolute discrepancy ($\bar{MAE}$ expressed in ms) between the muscle-wise water T2 gold standard values from EPG signal simulation algorithm and the predicted value through ML algorithms.

**Mean absolute discrepancy (** $\bar{MAE}$ **)**							
**Muscle**	**LR**	**Ridge**	**Lasso**	**TREE**	**RF**	**KNN**	**SVM**
S	4.31 (1.20)	3.92 (1.26)	3.85 (1.33)	4.66 (1.07)	3.63 (1.33)	2.36 (0.615)	3.59 (0.87)
MG	10.40 (1.26)	10.40 (1.22)	10.40 (1.18)	8.17 (1.64)	9.05 (2.36)	2.15 (0.34)	8.25 (2.02)
LG	13.23 (4.17)	9.49 (2.28)	4.73 (2.32)	8.08 (2.84)	9.02 (2.45)	6.14 (1.98)	7.90 (2.36)
TA	8.36 (1.07)	7.99 (0.90)	7.70 (0.74)	7.62 (1.19)	7.04 (1.15)	3.28 (1.07)	6.84 (1.23)
ELD	26.19 (47.84)	4.21 (1.43)	5.21 (2.20)	4.21 (1.70)	4.90 (2.01)	2.39 (1.51)	3.67 (2.16)
Pe	2.71 (1.03)	2.24 (0.75)	2.24 (0.82)	2.07 (0.56)	1.97 (0.70)	0.80 (0.38)	1.73 (0.71)

S.D. is reported in round brackets.

**Table 6:** Workflow 3: Evaluation of ML models predicting performances: mean absolute discrepancy ($\bar{MAE}$ expressed in ms) between the muscle-wise water T2 gold standard values from EPG signal simulation algorithm and the predicted value through ML algorithms.

**Mean absolute discrepancy (** $\bar{MAE}$ **)**							
**Muscle**	**LR**	**Ridge**	**Lasso**	**TREE**	**RF**	**KNN**	**SVM**
S	1.55 (0.45)	1.36 (0.45)	1.07 (0.45)	1.26 (1.33)	0.81 (1.17)	1.90 (0.58)	0.65 (0.97)
MG	8.46 (2.19)	8.46 (2.15)	8.46 (2.15)	9.26 (2.06)	10.40 (2.19)	2.06 (0.76)	8.00 (2.40)
LG	4.98 (0.69)	4.98 (0.73)	5.03 (0.69)	5.93 (0.90)	5.80 (1.16)	2.58 (0.99)	4.55 (1.57)
TA	9.91(2.58)	9.91 (2.54)	9.91 (2.50)	9.09 (2.91)	9.38 (2.62)	2.79 (1.07)	8.60 (2.66)
ELD	9.65 (3.47)	9.65 (3.47)	9.68 (3.20)	7.68 (2.01)	7.68 (2.05)	1.43 (0.502)	6.71 (2.35)
Pe	1.76 (0.25)	1.75 (0.24)	1.75 (0.24)	1.81 (0.27)	1.89 (0.30)	0.443 (0.15)	1.70 (0.27)

S.D. is reported in round brackets.

**Figure 5:**
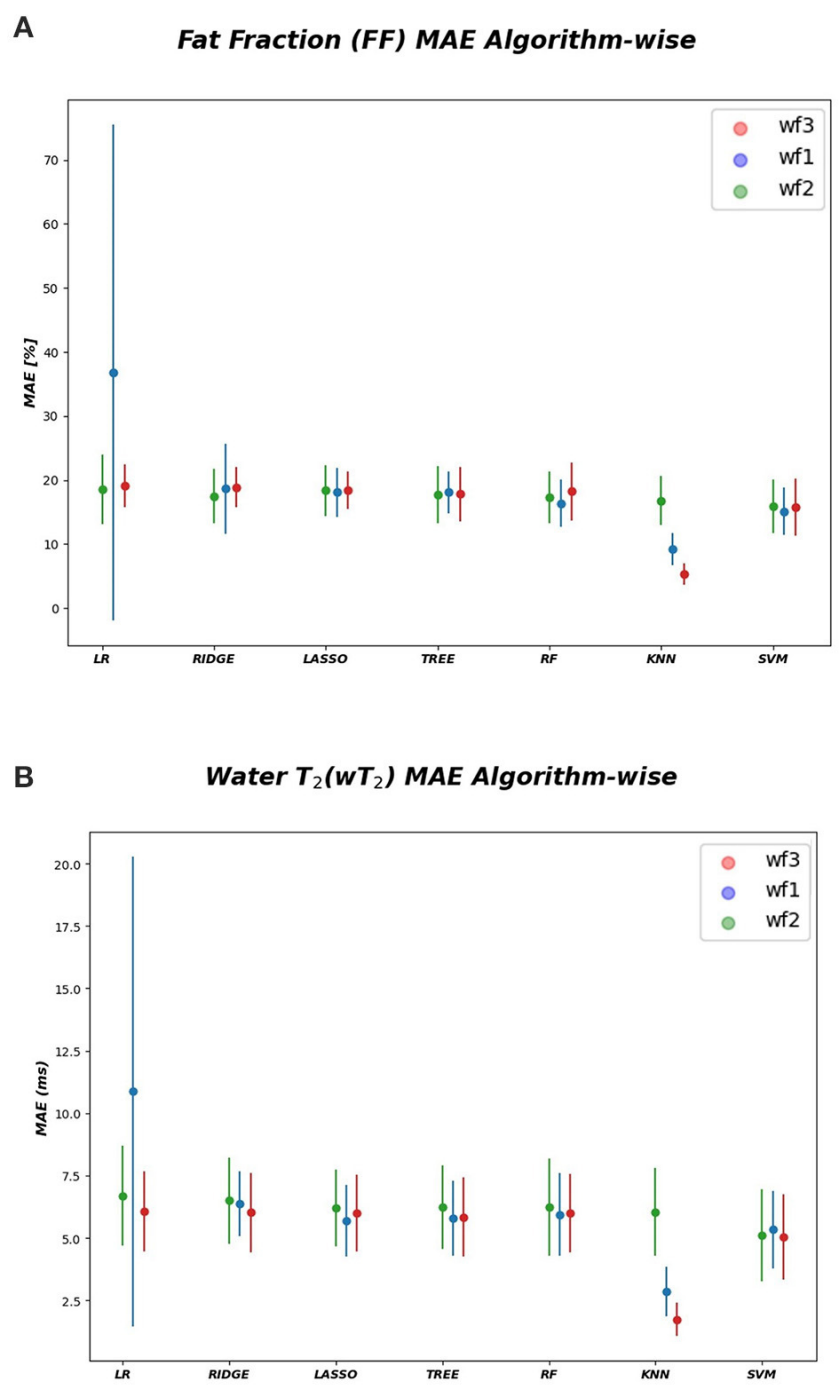
Algorithm-wise for FF and wT2. **(A)** FF (top) and **(B)** wT2 (bottom) prediction performances averaged on all muscles and showed as a function of the different implemented ML algorithms. According to the proposed workflows, a trio of mean prediction accuracy was defined for each ML model i.e., blue plot (WF1), green plot (WF2), red plot (WF3).

As inferred from boxplots in Figure 4, each workflow resulted in a mean FF and wT2 prediction performance of ± 20 *pp* and ± 6 *ms* (averaged values) for the anterior compartment muscles and of ± 15 *pp* and ± 6 *ms* for the posterior compartment, respectively. Figure 5 shows the mean prediction performance, averaged on all calf muscles, for each ML algorithm and workflow. KNN algorithm proved to be the best predictor model when combined with *WF3* for FF [$\bar{MAE}$± 5*pp* (*S.D*.1.8 *pp*)] and for wT2 [$\bar{MAE}$ ± 1.8 *ms* (*S.D*.0.7 *ms*)]. By contrast linear regression (LR) combined with *WF2* showed the worst accuracy in estimating FF [±36 *pp* (*S.D*.38.2 *pp*)] and wT2 [±10.9 *ms* (*S.D*.9.4)].

**Figure 6:**
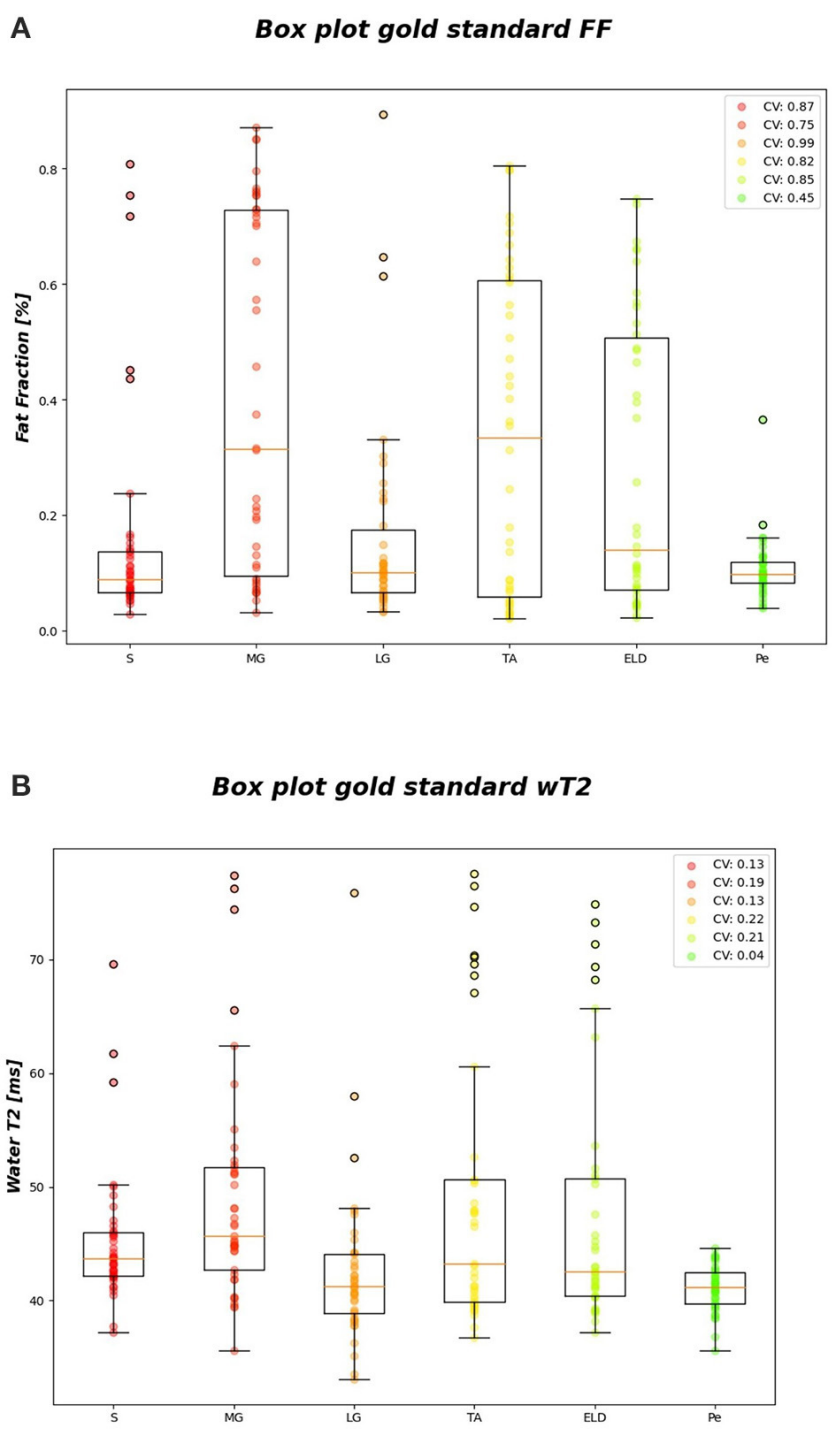
FF and wT2 gold standard boxplots. Muscle-wise boxplots (first quartile (Q1) to third quartile (Q3) and median value in orange line) **(A)** for FF (top) and **(B)** wT2 (bottom) gold standard values with CV listed in the legend.

**Figure 7:**
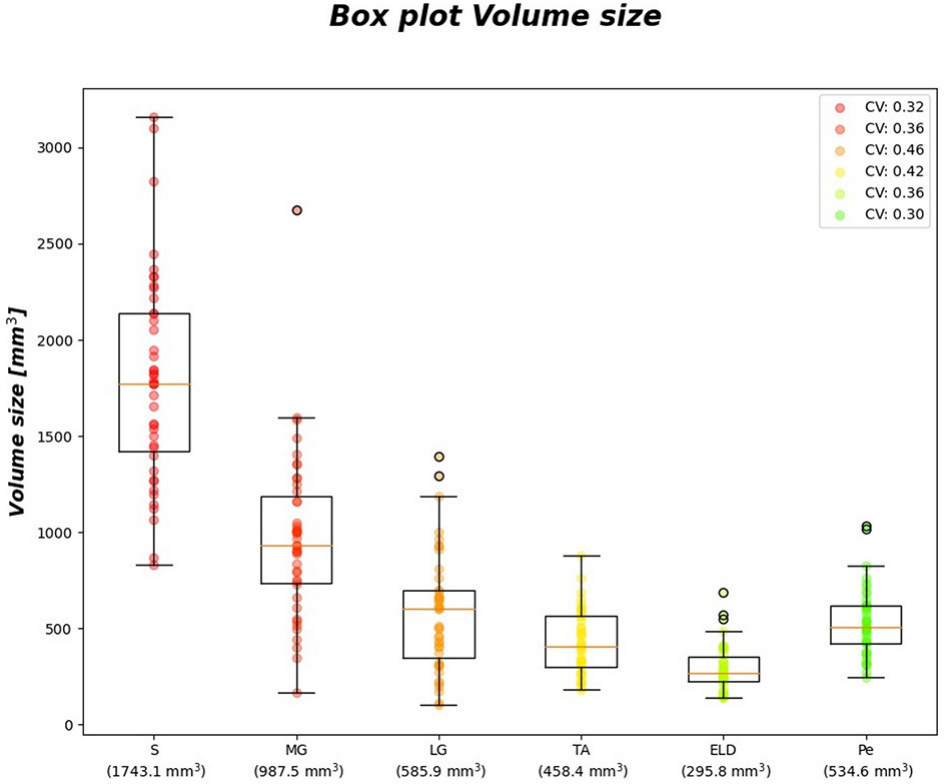
Volume size boxplots. Muscle-wise volume boxplots (first quartile (Q1) to third quartile (Q3) and median value in orange line). Muscle-wise mean volume size is reported in round brackets on x-axis, CV is listed in legend.

Figure 6 reports the *CV*_*i*_ for FF and wT2 for each calf muscle. Similarly, muscle volume CVs account for inter-subject muscle shape variability. Volume CVs are reported in Figure 7. The ground truth CVs range from 0.45 to 0.99 for FF and from 0.04 to 0.22 for wT2 whereas volume CVs range from 0.30 to 0.42 (Figures 6, 7).

Table 7 shows no significant correlation between KNN $\bar{MAE}\;$and both CVs of ground truth and volume values. Thus, KNN prediction seemed to be independent from inter-subject muscle shape, i.e., CVs volume, and ground truth variability ranges, i.e., CVs of FF and WT2. Furthermore, the presence of linear and monotonic correlations was tested even between KNN $\bar{MAE}$ and the mean volume of muscles to examine KNN prediction dependency on different calf muscle size. For our cohort, the following mean volume values for calf muscles were: *S* ≈ 1743.1 mm^3^, MG ≈ 987.5 mm^3^, LG ≈ 585.9 mm^3^, TA ≈ 458.4 mm^3^, ELD ≈ 295.8 mm^3^, Pe ≈ 534.6 mm^3^. Pearson and Spearman coefficients did not show any significant correlation neither for $\bar{MAE}$ FF [ρ_*P*_ = 0.66 (0.22) and ρ_*S*_= 0.52 (0.36)] nor for $\bar{MAE}\;$wT2 [ρ_*P*_ = 0.12 (0.83) and ρ_*S*_= 0.08 (0.87)]. Therefore, KNN prediction seemed to be independent even from dimension of calf muscles.

**Table 7:** Pearson and Spearman correlation coefficients between volume CVs, ground truths CVs and KNN $\bar{MAE}$ prediction of neuromuscular parameters.

**CV-parameter**	**Pearson**	**Spearman**
Vol-FF	0.19 (0.75)	0.10 (0.80)
Vol-wT2	0.75 (0.08)	0.71 (0.08)
FF-FF	0.43 (0.46)	0.58 (0.30)
wT2-wT2	0.65 (0.16)	0.55 (0.25)

*P*-values are reported in round brackets with a significant level set at *p* ≤ 0.05. KNN FF prediction for Pe muscle was not included to evaluate Pearson and Spearman correlations because it resulted to be an outliers of KNN FF $\bar{MAE}$ distribution.

### 3.1. Discussion

In this study, we explored the possibility to predict fat fraction and water T2 of calf muscles in FSHD subjects starting from a conventional STIR sequence and applying three different workflows, which combine radiomics, dimensionality reduction methods and ML models. To the authors' knowledge, this is the first attempt to predict qMRI parameters from STIR imaging, whereas MRI radiomics features extraction from STIR images have already been exploited to classify disease groups or autoantibodies in patients with idiopathic inflammatory myopathies (IIMs) with ML (42). The three applied workflows resulted in a comparable mean prediction performance about ± 20 *pp* for FF and about ± 6 *ms* for wT2 with the exception of LR and KNN models. KNN, according to the obtained results, turned out to be the best predictor model both for FF and wT2. More specifically, the algorithm-wise performance highlights the best prediction for the combination of KNN and *WF3* for both FF (±5 *pp*) and wT2 (± 1.8 *ms*). The muscle-wise analysis of the prediction performance also demonstrates a KNN mean prediction performance with almost no dependency either on the dimension of the muscles and on inter-subject muscle shape. We investigate these hypotheses by calculating for each muscle the muscle mean volume and the volume CVs. Despite the difference both in mean muscle-wise volume values and in volume CVs, no significant Pearson and Spearman correlation were found with KNN $\bar{MAE\;}$that was able to predict wT2 and FF with a mean error of approximately ± 1.8 *ms* and ± 5 *pp*, respectively.

Furthermore, the combination of a small sample size and high CV of ground truth distributions may have negatively affected the ML training step and consequently compromised prediction performance. However, KNN parameters prediction seemed to have no dependency on CV of ground truth values used for training ML algorithms. In contrast to the good predictive power of KNN, we found the least performative model being LR combined with *WF2*. We surmise that LR + *WF2* might be unable to detect the complex relationship between predictors and target variable as suggested by the wider error bars. The main limit of the current study is related to STIR sequence artifacts such as the low-signal-intensity banding artifacts and high-signal-intensity areas without proper fat suppression (43) that eventually may affect the FF prediction. Nevertheless, we used this non-uniform fat signal component to identify image fat pixels, which were used to extract conventional radiomics features (*WF1, WF2*), and to define FFG feature (*WF3*). Conversely STIR imaging is particularly suitable for muscle edema pattern detection (41) which may be easily detected by radiomic features. Furthermore, this study focused on the prediction by all WFs of the mean value of FF and wT2. FSHD is an asymmetric muscular dystrophy, therefore a more in-depth predictivity analysis that also takes into account the laterality of ROIs could be a useful tool for an ever-improving prediction model. Moreover, to expand the applicability of the current results, we aim to conduct further studies enrolling larger cohorts of subjects with different muscular dystrophies and also exploring other skeletal muscle districts (e.g., paravertebral muscles).

In conclusion, our study showed that conventional STIR imaging can potentially be used to predict quantitative muscle MRI parameters by applying radiomics combined with ML models. In particular, the KNN algorithm combined with WF3 was the best predictor for both FF and wT2. The proposed radiomic workflows could contribute to a wider application of a relatively common imaging technique as STIR to rapidly estimate quantitative parameters of skeletal muscle, without the need to acquire long and complex advanced qMRI sequences.

## Data availability statement

The data analyzed in this study is subject to the following licenses/restrictions. Requests to access these datasets should be directed to Direzione Scientifica, dirsci@mondino.it.

## Ethics statement

The studies involving human participants were reviewed and approved by the Ethics Committee of Pavia and the Ethics Committee of Fondazione Policlinico Universitario A. Gemelli. The patients/participants provided their written informed consent to participate in this study.

## Author contributions

GC and LB: conceptualization, methodology, software, and writing—review and editing. MP: data curation, supervision, and writing—review and editing. MM, ER, and GT: resources and writing—review and editing. NB: software and review. GM: data curation. XD and FS: resources, writing—review and editing, and supervision. AM and SF: validation, writing—review and editing, and supervision. AP: supervision, project administration, and writing—review and editing. All authors contributed to the article and approved the submitted version.
